# Detecting SARS-CoV-2 at point of care: preliminary data comparing loop-mediated isothermal amplification (LAMP) to polymerase chain reaction (PCR)

**DOI:** 10.1186/s12879-020-05484-8

**Published:** 2020-10-20

**Authors:** Marc F. Österdahl, Karla A. Lee, Mary Ni Lochlainn, Stuart Wilson, Sam Douthwaite, Rachel Horsfall, Alyce Sheedy, Simon D. Goldenberg, Christopher J. Stanley, Tim D. Spector, Claire J. Steves

**Affiliations:** 1 Department of Ageing & Health, Guy’s and St Thomas’ NHS Foundation Trust, London, UK; 2 Department of Twin Research and Genetic Epidemiology, Kings College London, Westminster Bridge Road, London, SE1 7EH UK; 3 MicrosensDx Ltd, 2 Royal College Street, London, UK; 4 Department of Infection, Guy’s and St Thomas’ NHS Foundation Trust, London, UK

## Abstract

**Background:**

A cost effective and efficient diagnostic tool for COVID-19 as near to the point of care (PoC) as possible would be a game changer in the current pandemic. We tested reverse transcription loop mediated isothermal amplification (RT-LAMP), a method which can produce results in under 30 min, alongside standard methods in a real-life clinical setting.

**Methods:**

This prospective service improvement project piloted an RT-LAMP method on nasal and pharyngeal swabs on 21 residents of a high dependency care home, with two index COVID-19 cases, and compared it to multiplex tandem reverse transcription polymerase chain reaction (RT-PCR). We recorded vital signs of patients to correlate clinical and laboratory information and calculated the sensitivity, specificity, positive predictive value (PPV) and negative predictive value (NPV) of a single swab using RT-LAMP compared with the current standard, RT-PCR, as per Standards for Reporting Diagnostic Accuracy Studies (STARD) guidelines.

**Results:**

The novel method accurately detected 8/10 RT-PCR positive cases and identified a further 3 positive cases. Eight further cases were negative using both methods. Using repeated RT-PCR as a “gold standard”, the sensitivity and specificity of a single novel test were 80 and 73% respectively. PPV was 73% and NPV was 83%. Incorporating retesting of low signal RT-LAMP positives improved the specificity to 100%. We also speculate that hypothermia may be a significant early clinical sign of COVID-19.

**Conclusions:**

RT-LAMP testing for SARS-CoV-2 was found to be promising, fast and to work equivalently to RT-PCR methods. RT-LAMP has the potential to transform COVID-19 detection, bringing rapid and accurate testing to the PoC. RT-LAMP could be deployed in mobile community testing units, care homes and hospitals to detect disease early and prevent spread.

## Introduction

Current diagnosis of COVID-19 relies on centralised laboratory-based RT-PCR (Reverse Transcription Polymerase Chain Reaction) testing. Although PCR provides a relatively rapid result, it is limited by the bottlenecks of transportation to the laboratory and the requirement to batch samples in a large run. Moreover, alternative technologies to RT-PCR requiring different reagents, and dry swabs would reduce the strain on laboratory and clinical supplies, allowing greater numbers of tests to be performed [1]. It is abundantly clear that urgent research is needed to enable health services globally to plan resources and this research must both move rapidly from bench to bedside and be scalable and rapidly available. In light of this urgency, we present a preliminary evaluation of a novel, quick test for COVID-19 that can be implemented at the point of need.

Point-of-care (PoC) testing may be critical to enable rapid detection of disease when an outbreak is suspected. This is particularly important in community settings like care homes, where multiple vulnerable patients reside together, and COVID-19 can spread quickly if not identified early [8]. Older residents are at higher risk of mortality from COVID-19 [9], and care homes have reported significant outbreaks both in the United Kingdom (UK) and internationally [10]. However, they have limited access to laboratory diagnostic services. A rapid, PoC test would allow early case identification, and implementation of increased infection control measures to prevent further spread to residents and staff, as recommended by The World Health Organization (WHO) [11] and British Geriatric Society [12]. Between 19th February and 8th April 2020, six independent groups have posted preprints of submitted manuscripts evaluating novel RT-LAMP testing methods against RT-PCR as gold standard (Table 1). Since then, a number [13] of other groups have published high-quality studies demonstrating that RT-LAMP has the potential to replace RT-PCR as a means for detecting SARS-CoV-2 (Severe acute respiratory syndrome coronavirus 2) within RNA extracted from nose - throat swabs and endotracheal secretions/bronchoalveolar lavage fluid [5, 14, 15].

**Table 1:** Articles comparing LAMP methods with RT-PCR for COVID-19 detection available at the time this study

Preprint	Country	Methods	Samples used for validation	Sensitivity of LAMP (for ORF1ab gene) compared with RT-PCR	Specificity of LAMP (for ORF1ab gene) compared with RT-PCR
El-Tholeth et al. [2]	USA	Two stage isothermal amplification (COVID-19 Penn-RAMP) targeting ORF1ab	No SARS-CoV-2 samples available in USA at time of study so samples with inactivated HIV virus with synthesised LAMP sequences tested. Four positive samples used.	75%	100%
Lamb et al. [3]	USA	LAMP using unspecified primers	No SARS-CoV-2 samples; synthesised LAMP sequences tested.	Study was not powered to determine sensitivity in a clinical population	N/a
Zhang et al. [4]	China	LAMP using ORF1ab, and N gene primers	6 positive swabs by RT-PCR	100%	N/a
Yu et al. [5]	China	LAMP using ORF1ab gene primers	43 positive swabs by RT-PCR	97.6%	N/a
Yang et al. [6]	China	LAMP using ORF1ab, E and N gene primers	208 swabs (17 positive & 191 negative by RT-PCR)	87.5% (confidence intervals not available)	99% (confidence intervals not available)
Yan et al. [7]	China	LAMP using ORF1ab, S-123 gene primers	130 specimens (both swabs and BAL specimens) – 58 positive, 72 negative	100%	100%

*BAL* Broncho-alveolar lavage, *USA* United States of America, *HIV* Human Immunodeficiency Virus

To this end, we used a combination of magnetic bead viral genome capture and optimised RT-LAMP (Reverse Transcriptase Loop-Mediated Isothermal Amplification) for amplification and detection of the SARS-CoV-2 genome; targeting the *ORF1ab* gene, to show proof of principle. The assay runs at 65 °C allowing simpler and cheaper instrumentation to be used with rapid results (< 25 min from swab to result). It can be used without a hospital laboratory and is suitable for a mobile testing unit model. Compared to RT-PCR, the method has a high sensitivity and specificity in laboratory evaluation [7] but is yet to be proven in clinical settings.

## Methods

### Study design

The setting was a National Health Service (NHS) high dependency care home (Category 1 Continuing Care), where an outbreak was suspected. All residents were eligible for inclusion. On Day 0 (Monday 16th March) two patients experienced fever and had other classical symptoms of COVID-19, arousing clinical suspicion. RT-PCR testing was performed on Day 1 and reported as positive on Day 2. To determine the extent of spread in the home, and protect patients and staff, on days 3 & 4 nasal and pharyngeal swabs were performed in all patients in the care home and analysed using multiplex tandem RT-PCR. On Day 4 a single RT-LAMP swab was used to sample the throat, followed immediately by the nose. Patients’ vital signs (including temperature, heart rate, blood pressure, respiratory rate and oxygen saturations) were noted in the 4 weeks before the known outbreak to determine whether the start of the outbreak may have occurred prior to the presumed day 0. Standards for Reporting Diagnostic Accuracy Studies (STARD) guidelines were used; STARD guidelines aim to improve the completeness and transparency of reporting studies of diagnostic accuracy, to allow readers to assess the potential for bias and to evaluate its generalisability [16].

### Test methods

In order to protect staff and patients, isolation and barrier nursing with full personal protective equipment were instituted for all patients. All patients were sampled on day 3 and day 4 using pharyngeal (Day 3) and deep nasal (Day 4) specimens (swabs) collected which were immediately placed into viral transport media (VTM) for RT-PCR or dry for the RT-LAMP assay. Staff taking the swabs were also swabbed and were negative for SARS-CoV-2 using RT-LAMP. Samples were urgently couriered to the hospital and MicrosensDx laboratory.

**Fig. 1:**
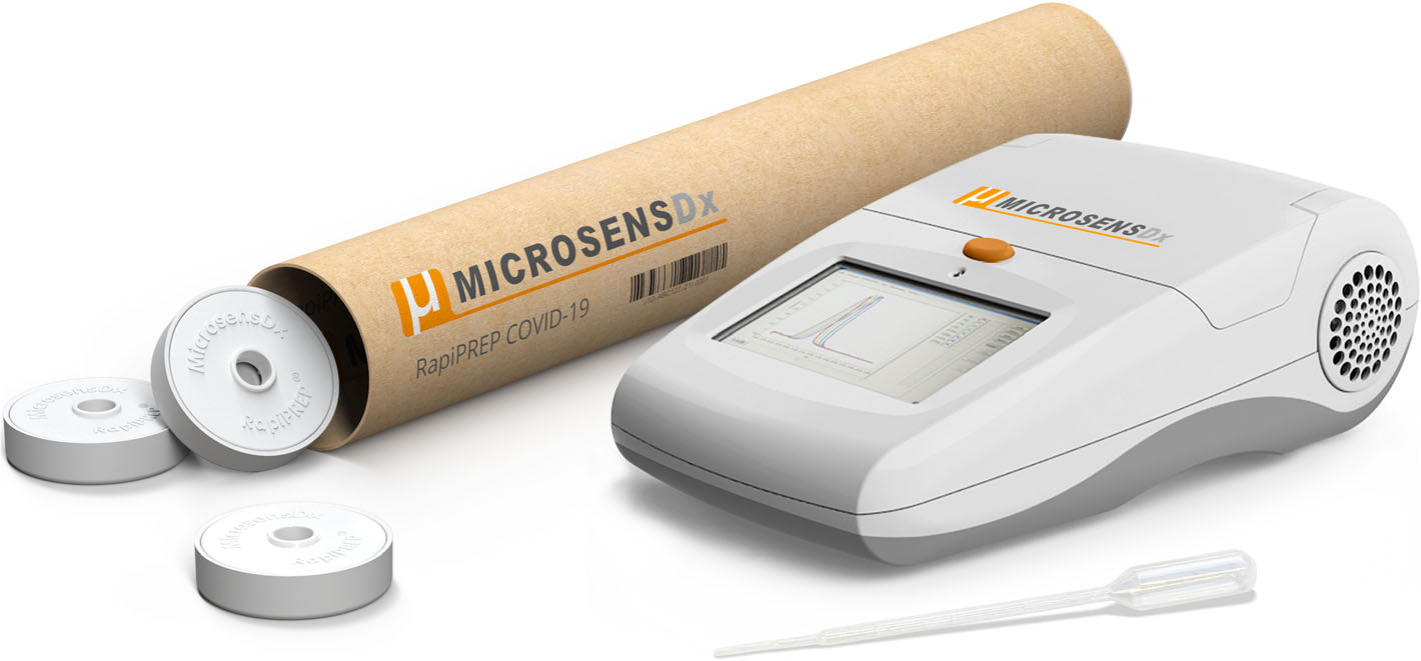
MicrosenseDx RapiPREP COVID-19 RT-LAMP System. Copyright: MicrosensDx, 2020 (Written permission granted for publication)

The hospital performed multiplex tandem RT-PCR according to standard protocols with the RT-PCR test targeting the *ORF1ab* gene only; the limit of detection of the RT-PCR was not determined by the lab or manufacturer, but for this technology it is typically < 80 copies per 10 μl nucleic extract input [17]. Input volume for RT-PCR was 200 μl of sample eluted to 60 μl, with just 10 μl of this used in the assay. If patients were positive on Day 3, Day 4 samples were not analysed, but have been stored for later analysis. The RT-LAMP method employed was the MicrosensDx RapiPrep® SARS-CoV-2 research use test (see Fig. 1). This method used magnetic bead capture to maximise the yield of target nucleic acid during sample preparation from the dry swab, which is followed by RT-LAMP to amplify and detect the SARS-CoV-2 genome, targeting the *ORF1ab* gene alone. The assay runs at 65 °C allowing simpler and cheaper instrumentation which can yield results in 25 min on average, often giving identification of positives in < 10 min. Results from this assay were compared to multiplex tandem PCR performed twice in the case of negative patients. Input volume for RT-LAMP was 40 μl of the RNA extract, which was the entire eluate from the magnetic bead extraction.

### Analysis

The sensitivity, specificity, positive predictive value and negative predictive value were calculated using Clopper-Pearson confidence intervals (CI) by comparing our Day 4 RT-LAMP result to RT-PCR. A patient was considered positive by RT-PCR if either a Day 1/3 result or a Day 4 test result was positive.

### Ethics, and patient and public involvement (PPI)

In view of the urgency of the COVID-19 pandemic and the need to act quickly in the outbreak, formal PPI consultation for this clinical improvement study was not possible. The study was discussed extensively with the care team and virology department and senior management. This project was a clinical service improvement and the requirement for research ethics committee (REC) approval was therefore waived in line with NHS Health Research Authority guidance (http://www.hra-decisiontools.org.uk/research/). In the spirit of participant involvement, the study was discussed with all capacitous patients in the care home. All were enthusiastic to be involved and could see the value of rapid testing. In addition, relatives of all the patients who lacked capacity were appraised of the study and given a chance to comment, and for their relative to not take part. One family felt an additional swab might be intrusive, but all others were keen to be involved, gave some suggestions about swab technique (nasal vs. pharyngeal) and for the results to be shared for the benefit of others.

## Results

**Fig. 2:**
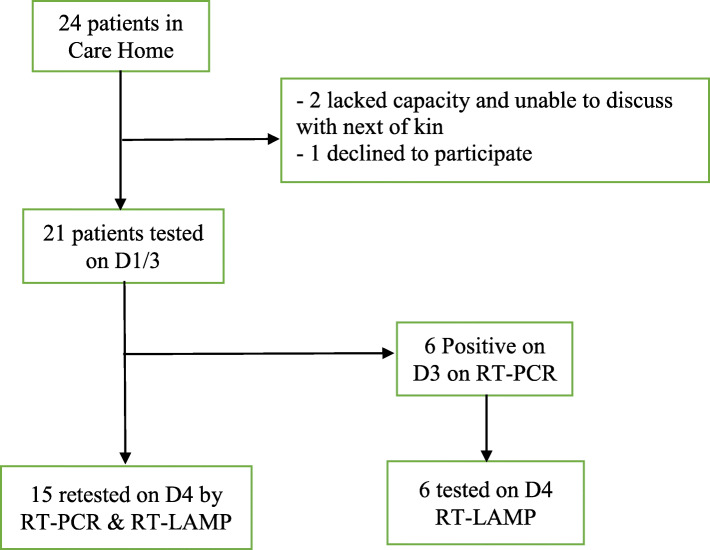
Flowchart of Patients

Twenty four residents were present in the care home on Day 0. Two patients lacked capacity and had no contactable next of kin to inform of the project. In one patient their informant did not agree to repeated testing as a service improvement. Twenty one patients were included in the study (Fig. 2). Study participants were aged between 52 and 89 years (median 76 years) and were predominantly female (70%). 2/21 died due to Covid-19, and 2/21 died from unrelated causes (for one patient, a progressive end-stage malignancy) within 7 days of their positive test (Table 2).

**Table 2:** Baseline Characteristics of Cohort

Age - Median	76 years
- Interquartile Range	61–81 years
Sex (Female)	17 (70%)
Any Fever (> 37.3C) in past 28 days	16/21
7-day Mortality - Due to COVID-19	2 (10%)
7-day mortality - Other Causes	2 (10%)

Testing results are shown in Table 3. We defined cases as being RT-PCR positive on one of two tests at day 3 or 4, and negative if negative on both tests. Using this definition, 10/21 patients in the facility were COVID-19 positive (RT-PCR34). Of these 10 cases, 8 were identified with a single swab for RT-LAMP, giving a sensitivity of 80% (95% CI 44–98%) and positive predictive value of 73% (95%CI 39–94%) (Table 3). This represented an improved rate of detection compared to single swab RT-PCR both in our sample and previous estimates.

**Table 3:** Participant results, vital signs, and mortality at 7 days

				Vital signs in 7 days prior to any positive result				
ID	D1/3 PCR (pharyngeal)	D4 PCR (nasal)	D4 LAMP (Nasal)	T > 37.8C	T < 36.0C	T > 37.3C	SpO2 < 92%	Death
1	–	–	–					
**2**	**+**	**n/a**	**+**	**Yes**			**Yes**	
3	–	–	–					
**4**	**+**	**n/a**	**+**	**Yes**				
**5**	**+**	**n/a**	**+**	**Yes**				**COVID-19**
**6**	**–**	**+**	**+**		**Yes**			
**7**	**+**	**n/a**	**+**					**(other cause)**
**8**	**+**	**n/a**	**+**	**Yes**				**COVID-19**
**9**	**–**	**+**	**+**		**Yes**			
10	–	–	–			Yes		
11	–	–	+	Yes				
12	–	–	+		Yes			
13	–	–	–					
14	–	–	–					
15	–	–	–					
16	–	–	–	Yes			Yes	
**17**	**–**	**+**	**–**	**Yes**			**Yes**	
18	–	–	+			Yes		
**19**	**–**	**+**	**–**					
**20**	**+**	**n/a**	**+**	**Yes**			**Yes**	
21	–	–	–			Yes		(other cause)

Rows for cases (defined as at least one of two positive RT-PCR tests) are highlighted in bold.

The specificity of the RT-LAMP test was 73% on a single test and 100% on retesting LAMP positives with low signal. Three cases were initially identified as low positive using RT-LAMP which were negative on RT-PCR, giving a total of 13 patients testing positive on either RT-PCR or RT-LAMP (Table 4). Of these 3 patients, patient 11 had a high grade temperature of 38.1 °C on D1 of testing, patient 12 had a temperature < 36.0 °C in the 7 days prior to testing and patient 18 had a temperature of 37.5 °C in the 7 days prior to testing (Table 3). All three remained well at day 10 with no other explanation for symptoms, such as upper respiratory or urinary tract infections. The routine test protocol now recommended by MicrosensDx includes retesting of low positive samples. Repeat RT-LAMP tests on samples from the three low positive patients were negative on repeat at day 9. It is possible that the RT-PCR results for one or more of these patients represent false negatives on day 4. Of the two patients positive for RT-PCR and negative using RT-LAMP one was contemporaneously symptomatic, and the other was well at the time of testing but had suffered a significant flu-like illness for the 3 weeks prior to Day 0.

**Table 4:** Testing results, comparing RT-LAMP method on deep nasal swabs to Multiplex tandem RT-PCR performed on both pharyngeal and deep nasal swabs

	PCR (repeat)		
	Positive	Negative	Total
LAMP			
Positive	8	0	8
Negative	2	11	13
Total	10	11	21
LAMP single test including low positives			
Positive	8	3	11
Negative	2	8	10
	10	11	21
			(95% CI)
Sensitivity single test		80%	(44–98%)
Specificity single test		73%	(39–94%)
Positive Predictive Value		73%	(39–94%)
Negative Predictive Value		80%	(44–98%)
Sensitivity – 2 LAMP tests		80%	(44 to 97%)
Specificity – 2 LAMP tests		100%	(72 to 100%)
PPV – 2 LAMP tests		100%	(n/a)
NPV – 2 LAMP tests		85%	(61 to 95%)

Positive PCR cases are defined as at least one positive test over 2 days.

**Fig. 3:**
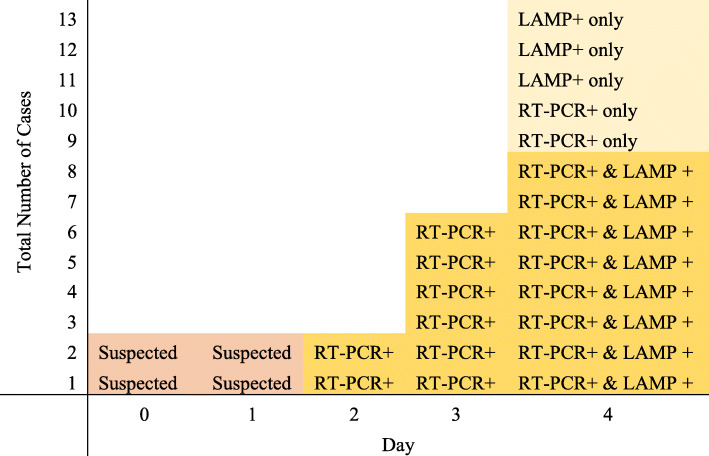
Development of the 13 cases and testing over time. Cases 1&2 were initial suspected index cases, confirmed by PCR on Day 2 and LAMP on Day 4. A further 4 cases were detected by universal PCR testing on Day 3, then LAMP on Day 4. On Day 4, 8 cases were confirmed by both universal RT-PCR and LAMP, with 2 new cases identified on RT-PCR only and 3 further cases on LAMP only (discrepant results in light yellow)

Many patients in the home had altered vital signs in the week leading up to testing, with 6/11 negative cases, and 8/10 positive cases showing signs, e.g. fevers or reduced oxygen saturations. Low temperatures (< 36 °C were detected in a minority of COVID PCR positive patients (Table 3). The development of cases in the home and testing results are illustrated in Fig. 3. No adverse events related to testing were reported.

## Discussion

In a time of global crisis, it is critical that data are quickly shared on new testing methods, so that they can be scaled up more rapidly. To this end, we present data from 21 patients in a care home tested within days of an outbreak in the home.

In this patient group, a single RT-LAMP test had a sensitivity of 80% and a specificity of 73% on single test compared to a “better than gold standard” of two consecutive RT-PCR swabs. The specificity of the RT-LAMP improved to 100% when the new protocol of retesting low positive LAMP tests is performed. We feel that this level of sensitivity is “clinically workable” in a time of crisis, particularly if repeated testing is utilised, and safeguards are put in place to guard against overconfidence in negative individuals, but this is somewhat subjective as no defined threshold of acceptability exists. It is comparable to other estimates of a single-swab RT-PCR test in our clinical experience and in posted pre-prints [18, 19]. Combined with the rapid result time, RT-LAMP may have additional clinical utility to standard RT-PCR. The RT-PCR negative, RT-LAMP initial low positive samples may indicate a lack of specificity of the low-level RT-LAMP signals. Given that some infected patients are assumed to be have been clinically asymptomatic and given that the RT-LAMP assay used here tests more of the swab eluate than the PCR, these may be real positive results at day 4, that have missed by the RT-PCR. Further testing and further studies will resolve this issue.

In addition, we found fever > 37.3 °C, as expected was a common symptom, but hypothermia (T < 36.0 °C) and desaturation were also noted. The finding of hypothermia is important. It is a recognised symptom of sepsis and the systemic inflammatory response syndrome, particularly in older people [20]. However, current PHE and WHO COVID-19 guidelines do not include hypothermia as a symptom. Larger scale studies on prevalence of hypothermia, as well as other non-classical symptoms, would shed more light on the presentation of COVID-19 in institutionalised patients.

Loop-mediated isothermal amplification (LAMP) was developed as a rapid and reliable, cheaper method to amplify from a small amount target sequence at a single reaction temperature, obviating the need for sophisticated thermal cycling equipment [21]. Two of these used only proven PCR-positive throat and nasal swabs and demonstrated sensitivity > 97% for RT-LAMP methods targeting the *ORF1ab* gene when compared with gold standard RT-PCR [4, 5]. Only the studies by Yang [6] and Yan [7] included samples from both SARS-CoV-2 positive and negative patients and was thus able to produce both a sensitivity and a specificity. The remaining two groups, both based in the United States, lacked access to, or clearance to work with, SARS-CoV-2 samples and used either inactivated HIV with synthesised LAMP sequences [2] or other synthesised RT-LAMP sequences [3]. The majority of studies focused on the highly-conserved *ORF1ab* gene primer, also targeted by the RT-LAMP method used by the MicrosensDx RapiPrep® SARS-CoV-2 method. Our study is the first “real world” study comparing the effectiveness of RT-PCR and RT-LAMP testing in a group of patients at high risk for COVID-19 and represents an important progression to clinical use for this novel SARS-CoV-2 testing method. We planned to perform RT-LAMP testing just once due to a high degree of confidence that a single test would have satisfactory accuracy, allowing clinical decisions to be made immediately. However, the 3 discrepant samples were fully concordant on re-test. As such, our standard for comparison was not a single RT-PCR, but two separate swabs for RT-PCR sent on consecutive days, thus representing what could be considered a “better-than gold standard” for comparison. However, as the pandemic has progressed, it has become apparent that there is no true “gold standard” for COVID-19 testing with highly-anticipated antibody testing not always proving helpful; even in mild disease, antibodies in PCR positive patients may not be detected [22].

We have been able to perform these tests quickly in a group at high risk for severe disease, and a setting where early identification of infected patients is key to preventing further spread. Many other studies so far have used laboratory samples to estimate efficacy but have been unable to estimate the clinical utility. Swabs were taken by the same clinician, minimising the risk of technical error or observer biases. All RT-LAMP samples were tested in the MicrosensDx laboratory, and RT-PCR in the hospital laboratory, and there was no viral transport medium on the RT-LAMP swabs. Our samples were shipped to MicrosensDx because a Level 2 Biosafety cabinet was available in the company’s laboratory for initial sample handling and, due to the urgency of the study, there was not time to install a suitable cabinet in the care home. In the future a PoC facility may still require a Level 2 cabinet, however recent developments in sample collection devices that inactivate the virus immediately after swabbing are expected to eliminate the need for operator protection and so a biosafety cabinet will not be required.

Actual PoC testing, and or viral medium could be used to optimise performance further but use of dry swabs could ease issues with supply of viral transport media. We are limited by a small sample size, so our estimates have wide confidence intervals. However, they appear to be concordant with other (pre-print) studies on RT-LAMP performed purely on laboratory samples.

Cost is a significant issue when large-scale testing within the setting of a pandemic is considered. The combined sample preparation and LAMP assay kit at list price from MicrosensDx is equivalent in cost to a separate sample extraction kit and PCR test kit used in the reference laboratory. However, the LAMP instrument is significantly cheaper than a PCR machine (by a factor of 2-3x) providing a cost saving. Subsequent technology developments in the LAMP assay since this study was performed early in the pandemic include conversion to a colorimetric signal that can be read by eye, potentially negating the need for instrumentation altogether. Additionally, LAMP assays are currently being trialled with saliva.

Use of this rapid test could facilitate early identification of cases and enactment of infection control measures as required. We speculate that this could significantly reduce spread and subsequent mortality in care home residents, a speculation which could easily be tested if the method was more widely available. The test may also be suitable for use in other community settings such as pharmacies and care agencies, as well as emergency departments, and prisons or residential settings for homeless people where rapid diagnosis would be most useful. An area of global concern is COVID-19 spread in developing countries, where reported cases are increasing. Inexpensive PoC testing that is not dependent on skilled and centralised technicians will be vital for less well-resourced countries and economies. However, evaluation in these settings would be advised to replicate its effectiveness.

## Conclusion

There is an urgent need for a rapid, robust and cost-efficient POC test that can be used in care homes, community settings and away from centralised large-scale laboratories, without the need for skilled technicians. Magnetic bead capture and RT-LAMP amplification and testing for SARS-CoV-2 was found to be promising, rapid, easy to use and to work equivalently to standard multiplex tandem PCR methods. Definitive studies to evaluate this method in larger cohorts are underway. RT-LAMP has the potential to transform COVID-19 detection, bringing rapid and accurate testing to the PoC.

## Data Availability

The datasets used and/or analysed during the current study are available from the corresponding author on reasonable request.

## Abbreviations

CI: Confidence Interval
HIV: Human Immunodeficiency Virus
LAMP: Loop-mediated isothermal amplification
NHS: National Health Service
NPV: Negative Predictive Value
PCR: Polymerase Chain Reaction
PoC: Point of Care
PPI: Patient and public involvement
PPV: Positive Predictive Valvue
REC: Research Ethics Committee
RT-LAMP: Reverse Transcription Loop mediated isothermal Amplification
RT-PCR: Reverse transcription Polymerase Chain Reaction
SARS-CoV-2: Severe Acute Respiratory Syndrome Coronavirus 2
STARD: Standards for Reporting Diagnostic Accuracy Studies
UK: United Kingdom
USA: United States of America
WHO: World Health Organisation
